# Supplementary material for: Trajectory patterns and factors influencing perinatal fatigue among Chinese women from late pregnancy to 6 months after delivery
Source: PeerJ. 2022 May 26;10:e13387. doi: 10.7717/peerj.13387 (PMC9148558; doi:10.7717/peerj.13387)
Supplement: Supplemental Information 3 [file peerj-10-13387-s003.docx]

**Supplemental Table2.**

**Basic information between included and non-included subjects.**

| **Variables** | **Included Subjects**  **(N=1030)** | **Non-included Subjects**  **(N=120)** | **p** |
| --- | --- | --- | --- |
| Age, m(SD), y | 30.43(3.65) | 31.03(3.02) | 0.084 |
| Parity, n(%) |  |  | 0.114 |
| Primipara | 778(75.8) | 83(69.2) |  |
| Multipara | 249(24.2) | 37(30.8) |  |
| Employment, n(%) |  |  | 0.264 |
| Employed | 811(80.9) | 98(85.2) |  |
| Unemployed | 191(19.1) | 17(14.8) |  |
| Education Level, n(%) |  |  | 0.424 |
| <college | 80(7.9) | 7(5.9) |  |
| college | 191(18.9) | 18(15.3) |  |
| >college | 742(73.2) | 93(78.8) |  |
| Monthly family Income, n(%) |  |  | 0.085 |
| <￥10,000 | 130(12.9) | 8(6.7) |  |
| ￥10,000~20,000 | 502(50.0) | 58(48.7) |  |
| >￥20,000 | 373(37.1) | 53(44.5) |  |
| Residency, n(%) |  |  | 0.367 |
| Urban | 984(96.9) | 118(98.3) |  |
| Suburban | 32(3.1) | 2(1.7) |  |
| Planned Pregnancy, n(%) |  |  | 0.078 |
| Yes | 705(69.7) | 93(77.5) |  |
| No | 306(30.3) | 27(22.5) |  |
| Fetus Gender, n(%) |  |  | 0.556 |
| Boys | 464(51.6) | 56(48.7) |  |
| Girls | 435(48.4) | 59(51.3) |  |

*Footnotes*. m=mean, SD=standard deviation, n=number, %=percentage, y=year(s).
